# Muscle power is associated with higher levels of walking capacity and self-reported gait performance and physical activity in individuals with cerebral palsy

**DOI:** 10.3389/fphys.2024.1488905

**Published:** 2025-01-06

**Authors:** Mattie E. Pontiff, Abhinandan Batra, Li Li, Noelle G. Moreau

**Affiliations:** ^1^ Center of Innovation for Veteran Centered and Value Driven Care, Rocky Mountain VA Medical Center, Aurora, CO, United States; ^2^ Department of Physical Medicine & Rehabilitation, University of Colorado Anschutz Medical Campus, Aurora, CO, United States; ^3^ Department of Physical Therapy, University of Louisiana- Monroe, Monroe, LA, United States; ^4^ Department of Health Sciences and Kinesiology, Georgia Southern University, Statesboro, GA, United States; ^5^ Department of Physical Therapy, Louisiana State University Health Sciences Center- New Orleans, New Orleans, LA, United States

**Keywords:** muscle power, walking, activity, participation, gait speed, muscle performance

## Abstract

**Introduction:**

The purpose of this study was to investigate the relationships between a Power Leg Press test (PLP) with walking capacity and self-reported performance and participation in individuals with Cerebral Palsy (CP), and to compare the strength of the associations between two power tests (PLP and isokinetic (IsoK)) with walking capacity.

**Methods:**

Ambulatory individuals with CP (n = 33; age 17.89 ± 7.52 years) performed five inclined power leg presses at 40%–50% of their 1-repetition maximum “as fast as possible”. A linear position transducer was attached to the weight bar, and the displacement, total load, and angle of the sled were used to calculate peak power for each trial. Isokinetic knee extensor power was measured at 60 deg/sec. Walking capacity was measured using the 10-m walk test fast (FS) and self-selected (SS) speeds and the 1-min walk test (1MWT). Self-reported performance and participation measures were the Activities Scale for Kids-performance (ASKp), Patient-Reported Outcomes Measurement Information System (PROMIS^®^), and the Gait Outcomes Assessment List (GOAL). Pearson’s correlation coefficients determined relationships between power measures with walking capacity and self-report measures (α < 0.05).

**Results:**

PLP and IsoK power were significantly correlated to SS (r = 0.361, r = 0.376), FS (r = 0.511, r = 0.485), and 1MWT (r = 0.583. r = 0.443), respectively (*p* < 0.05). There was no significant difference between the strength of the associations between walking capacity and each test of power (PLP and Isok) (*p* > 0.05). PLP power was significantly correlated to composite scores on the ASKp (r = 0.690) and GOAL (r = 0.577) and to four components of the PROMIS, including physical function (r = 0.588) (*p* < 0.01). The Gait and Mobility subscale of the GOAL (r = 0.705) and the Locomotion (r = 0.636), Transfers (r = 0.547), and Standing (r = 0.521) subscales of the ASKp had strong relationships to peak power produced during the PLP test (*p* < 0.01).

**Discussion:**

PLP power was significantly correlated with walking capacity and self-reported walking performance and mobility-based participation in ambulatory individuals with CP. Higher movement velocities associated with the PLP test may explain the significant associations of power with faster gait speeds. Self-reported mobility performance and physical activity also showed moderate to strong relationships with lower extremity power. Overall, these results suggest a strong link between decreased muscle power generation and walking limitations in individuals with CP.

## 1 Introduction

Cerebral Palsy (CP) is a neurologic disorder resulting from injury to the brain prior to or shortly after birth (Rosenbaum et al., 2007) leading to significant and lasting impairments to the neuromuscular system including decreased muscle performance (weakness, decreased power and decreased rate of force development), spasticity and co-contraction (Damiano et al., 2001; Ross and Engsberg, 2007; Moreau et al., 2012; Moreau, 2013). Impairments in muscle performance have devastating impacts on individuals with CP as they influence walking and other critical mobility skills that are essential for maintaining independence and quality of life (Lundh et al., 2018). The World Health Organization’s International Classification of Function and Disability (ICF, 2001) model is a biopsychosocial model that can be used to organize and describe the influence CP has across three domains and how these domains influence each other (2001). The three domains include: 1) body systems (parts of the body and their function), 2) activities (both *capacity* and *performance* of tasks by an individual), and 3) participation (function of an individual in all areas of life). For example, in those with CP, lower extremity muscle weakness (impairment in body system domain) limits walking ability (limitation in activity domain) which may result in difficulty participating in school and recreational tasks (participation restriction) (Pirpiris et al., 2003; Schenker et al., 2005; Morgan et al., 2014). Thus, understanding the muscle performance impairments which are most impactful on walking ability will be important in developing rehabilitation interventions that are functionally meaningful for those with CP.

Muscle strength and power are two elements of muscle performance that are critical to walking activity and participation in those with CP. Lower extremity muscle strength, or the ability to produce maximal force, is decreased in those with CP (Wiley and Damiano, 1998) and is associated with greater walking capacity in individuals with CP (Damiano et al., 2001). Muscle power is defined as the product of force and velocity and is more impaired than muscle strength in those with CP (Moreau, 2013; Moreau et al., 2013; Geertsen et al., 2015). In addition, high velocity (power) training has been shown to be effective at improving walking speed and functional walking performance in youth with CP (Moreau et al., 2013; Kara et al., 2023). Only one study has explored associations between power and walking capacity (Moreau and Lieber, 2022) and none have reported associations between power and self-reported activity and participation. Moreau et al. reported a moderate to good relationship between isokinetic knee extensor power with both fast gait speed (r = 0.65; *p* < 0.001) and the 1-min walk test (1MWT) (r = 0.79; *p* < 0.001) in a cohort of ambulatory individuals with CP (Moreau and Lieber, 2022), however this study used an isokinetic measure of power and evaluated these relationships retrospectively. To our knowledge, no studies have prospectively examined the associations between power and walking activity and participation in those with CP. Thus, additional studies are needed to understand how muscle power influences individuals with CP across the ICF continuum of activity (capacity and performance) and participation.

Muscle power is typically measured using isokinetic dynamometry, cycle ergometry, or with clinical field tests. While each of these measures have value, they present several clinical limitations. Isokinetic dynamometry, the gold-standard measure of muscle power, is costly and can be time consuming to set up and administer which can limit clinical feasibility. Similarly, cycle ergometry tests, like the Wingate Anaerobic Test (WAnT), require specialized computer equipment to measure lower extremity power which can also be cost prohibitive in most clinical settings (Bar-Or, 1987). Most recently, Verschuren et al. developed a field test to measure power, the Muscle Power Sprint test (MPST) (Verschuren et al., 2007). While this test is cost effective and clinically feasible, it requires individuals with CP to sprint which has limited use in those with lower mobility levels. Based on current evidence, there are few tests of lower extremity power that are clinically feasible, cost effective, and appropriate for individuals with CP with a wide range of mobility levels (Pontiff et al., 2021; Pontiff et al., 2023).

A power leg press (PLP) test was recently shown to be a valid and reliable measure of lower extremity power in typically developing individuals (Pontiff et al., 2021) as well as ambulatory adults and children with CP (Pontiff et al., 2023). The PLP test measures lower extremity muscle power during a closed-chain leg press activity on a standard piece of fitness equipment. Further, the PLP test also uses an adjustable inclined leg press and weight bar attachment to accommodate a wide range of mobility levels in those with CP. Thus, the PLP may be a useful test as it is clinically feasible, more cost-effective than isokinetic dynamometry, and adaptable to wide range of mobility levels in individuals with CP. However, associations to key functional tasks like walking capacity and participation have not been explored. Further, the strength of the associations to walking capacity in comparison to the gold standard measure of lower extremity power, isokinetic dynamometry has not been evaluated. Understanding the strength of these relationships will aid researchers and clinicians in selecting the measure of power with the closest association to function which can be used to measure change in patient performance after intervention.

Thus, the first aim of this study was to examine how lower extremity muscle power produced during a closed chain PLP test was associated with walking capacity and self-reported walking performance and participation in individuals with CP. We hypothesized that lower extremity muscle power will be positively correlated to tests of walking capacity and self-reported performance and participation. The second aim of this study was to compare the strength of the relationships between muscle power produced during the PLP test and a gold standard test of power, isokinetic dynamometry, with walking capacity. For the second aim, we hypothesized that the PLP test will demonstrate stronger relationships to walking capacity compared to isokinetic dynamometry because the PLP test is a closed chain movement requiring use of the hip and knee extensors as well as the plantarflexors, which are the same critical muscle groups used in gait. The isokinetic power test is an open chain task that isolates the knee extensors. Using clinically feasible power tests to explore key relationships between muscle power with walking capacity and self-reported activity and participation will guide rehabilitation providers and researchers in the development of important exercise interventions to target improvements in activity and participation for those with CP.

## 2 Materials and methods

Ambulatory adults and children with a diagnosis of CP (Gross Motor Function Classification System level I-III) between 10–40 years were recruited and eligible for participation in this prospective study. Participants were excluded if they met the following criteria: botulinum toxin injections in the lower extremities in the last 3 months, knee flexion contractures >25°, surgery on the lower extremities in the last year, or inability to ambulate. This study was approved by a university Internal Review Board (IRB). Study participants were recruited from physical therapy clinics, physician referral, community organizations, and from a randomized clinical trial (Clinicaltrials.gov: NCT 03625570). Informed consent and assent were obtained prior to testing from adult and child participants, respectively.

### 2.1 Testing procedures

Following consent, participants completed a brief health and surgical history in addition to a short physical exam which included: height, body mass, and leg length measures.

### 2.2 Power leg press (PLP) test

Specific testing procedures for the PLP test have been previously published (Pontiff et al., 2021; Pontiff et al., 2023), but a brief description is provided here. 1-Repetition Maximum (1-RM) testing was performed prior to the PLP test to determine the appropriate load for the power test. Methods for 1-RM testing for individuals with CP have been previously published (Pontiff and Moreau, 2022). Power testing loads were set at 40%–50% of the individual’s 1-RM based on the testing guidelines for healthy adults (ACSM, 2019) and children (Faigenbaum et al., 2009). To begin, participants were positioned on the Total Gym^®^ (Total Gym Fitness Studios, San Diego, CA) leg press with their knees flexed to 90° (measured with goniometer), feet flat on the foot plate and the hands on the handlebars with the elbows fully extended. This starting position was maintained for both the 1-RM and PLP test. Participants performed three to four practice trials where they pushed the load “as fast as possible” from 90 degrees of knee flexion to their full knee extension and returned slowly to the start position. Participants rested for 5 minutes and then performed five consecutive power leg presses. A linear position transducer (TE Connectivity^®^, SGD 120-in Cable Actuated Sensor; Chatsworth, CA) was attached to the weight bar. Displacement of the transducer cable during the concentric phase of the movement produced a voltage signal (sampling frequency 500 Hz) that was recorded and converted into position-time data by a custom LabVIEW^®^ (National Instruments Corp., Austin, Tx) program. Power was calculated as the product of force and velocity using custom MATLAB^®^ (Mathworks Inc., Natick, MA) code. Force (*F*) was defined as F = m*g*sin *(θ)*, where *m* is the total mass of the system (body mass + external weight plates + mass of the sled in kg), *g* is acceleration due to gravity (9.81), and θ is equal to the angle of the leg press sled from the horizontal (29.4°) (Pontiff et al., 2021). Peak Power was calculated as the maximal power value in the concentric phase of the press. The mean of all valid trials from the test was normalized to participant body mass in kg and this value was used for all subsequent analyses. We selected mean peak power across trials because this variable was the most reliable and had the smallest minimal detectable change (MDC) in children and adults with CP compared to three other power measures in our validation study (Pontiff et al., 2023). Overall, measures of peak power had smaller MDC scores as compared to measures of average power for the PLP test (Pontiff et al., 2023). Repetitions were removed if the participant demonstrated a movement error or reduced effort as indicated by a value below 2SD of the mean of the five repetitions.

### 2.3 Isokinetic dynamometry

An isokinetic dynamometer (System 4, Biodex Medical Systems, Shirley, NY) measured knee extensor muscle power of the participants most involved limb at 60°/second. Participants were seated upright (trunk 85° from the horizontal) with their knee joint aligned with the axis of the dynamometer. The trunk, waist, and thighs were secured with straps, and the arms were crossed across the chest to minimize compensatory movements during testing. The range of motion of the knee was set through the full available passive range, and torque was gravity corrected. One to three practice trials were performed for warm up and familiarization. Following practice, each participant rested for 5 minutes.

For the isokinetic (IsoK) test, participants were asked to kick their leg into extension “as hard and as fast as possible” for five repetitions. Consistent verbal encouragement was given for each repetition. IsoK Power (IsoK P) was defined as the highest average power produced across the five repetitions during the concentric constant velocity portion of the knee extension movement at 60°/second, which was calculated by the computerized software of the isokinetic dynamometer (System 4, Biodex Medical Systems, Shirley, NY). Repetitions were removed if the participant demonstrated a movement error or reduced effort as indicated by a value below 2SD of the mean of the five repetitions at 60°/second. IsoK power data were normalized by body mass in kg for statistical analyses.

### 2.4 Measures of activity: walking capacity tests

Walking capacity was measured using the 10-m walk test (Fast and Self-selected: FS and SS speeds) and the 1-min walk test (1MWT). Participants wore comfortable clothes, walking shoes, braces or orthoses (if appropriate), and used any assistive devices they used for community walking.

For the 10-m walk test, participants were instructed to walk on a flat, level 14-m course in a hallway. The middle 10 m was timed for each test, so a 2-m acceleration and 2-m deceleration were marked to indicate where the timer was to start and stop, respectively. Participants were instructed to “walk at your normal comfortable walking speed until you reach the end of the hall” for the SS test. For the FS speed, individuals were instructed to “walk as fast as you safely can until you reach the end of the hall”. Three trials were performed at both speeds, and the average time for three trials was divided by 10 m to calculate a FS and SS gait speed in meters per second (m/s). The 10-m walk test has been shown to be a valid and reliable measure of gait speed in those with CP (Graser et al., 2016; Bahrami et al., 2017).

Following the 10-m walk test, participants rested for 5 minutes and were given instructions for the 1MWT. Participants then walked as fast as possible without running for 1 min. Each participant was notified when there were 30 s, 10 s and 5 s remaining. When time expired, participants were instructed to “stop” and the floor was marked with tape of the farthest forward foot and the score was recorded as meters walked in 1 minute. The 1MWT has been shown to be a valid and reliable measure of walking capacity in individuals with CP (McDowell et al., 2009).

### 2.5 Measures of self-reported activity (performance) and participation: self-report questionnaires

The Performance version of the Activities Scale for Kids (ASKp), Patient-Reported Outcomes Measurement Information System (PROMIS^®^)-Pediatric Profile-49 v2.0, and the Gait Outcomes Assessment List (GOAL) were used to measure self-reported performance and participation. Parent and participant versions were used for the PROMIS and GOAL as appropriate. The ASKp is a 30-item self-reported measure with nine sub-domains (Young et al., 2000). Scores are expressed as a percentage of function with lower scores indicating less functional ability. The PROMIS Pediatric and Parent Proxy Profile instruments are a collection of six self-reported questionnaires with the following domains: Depressive Symptoms, Anxiety, Physical Function-Mobility, Pain Interference, Peer Relationships, along with a single item on Pain Intensity (DeWalt et al., 2015). The PROMIS is a valid and reliable measure for children with CP and the mobility domain is capable of distinguishing between Gross Motor Function Classification System (GMFCS) levels (Kratz et al., 2013). The GOAL is a 48-item self-reported questionnaire with seven domains that evaluate a child’s performance related to gait function and mobility. The GOAL can discriminate between GMFCS levels and is a valid measure of gait function in ambulant children with CP (Thomason et al., 2018).

### 2.6 Statistical analysis

We used a pilot sample to perform an *a priori* power analysis. Based on a sample of 15 youth with CP, isokinetic knee extensor power was strongly associated with distance walked during the 1MWT (r = 0.78, *p* < 0.05). Assuming similar results for Aim 1, we determined that a minimum sample size of 30 achieves 90% power to detect a difference of 0.55 between the null hypothesis correlation of 0, and the alternative hypothesis correlation of 0.55 using a two-sided hypothesis test. For Aim 2, assuming that we will obtain a similar r value, a sample size of 30 would be enough to detect a difference of 0.16 between two tests’ correlation coefficients with 82% power. Power increases to 100% when the difference is greater than 0.16.

The relationships between each power test (PLP and IsoK) with tests of walking capacity and self-reported activity and participation measures were examined using Pearson’s correlation coefficients. Correlation coefficients were categorized based on previously published recommendations with values < 0.25 considered poor, 0.25–0.50 were weak, 0.5 to 0.75 were moderate to good, and coefficients >0.75 were excellent associations (Portney LG, 2000). Correlation coefficients were compared statistically to determine which test (PLP or IsoK) demonstrated a stronger relationship to walking capacity. To compare correlation coefficients, we used methods published by Steiger (1980). A computerized software program (Lee, 2013) converted correlation coefficients to z-scores using Fisher’s transformation and the asymptotic covariance of the estimates were used for an asymptotic z-test to determine if the correlations were statistically different from one another. Significance levels were set at 0.05 for all analyses.

## 3 Results

Thirty-three individuals with CP participated in this study. Participant demographics are reported in Table 1.

**TABLE 1:** Participant demographics.

Characteristics	All participants (n = 33)
Sex	
Male	12
Female	21
Age	
mean (SD), yrs	17.89 (7.52)
range, yrs	10–37
Height, mean (SD), m	1.52 (0.13)
Body Mass mean (SD), kg	51.22 (15.49)
Topographical Classification, n	
Hemiplegia	4
Diplegia	22
Triplegia	3
Quadriplegia	4
GMFCS Level, n	
I	8
II	22
III	3

Abbreviations: Gross Motor Function Classification System (GMFCS) level.

### 3.1 Associations between lower extremity power and walking capacity

**FIGURE 1:**
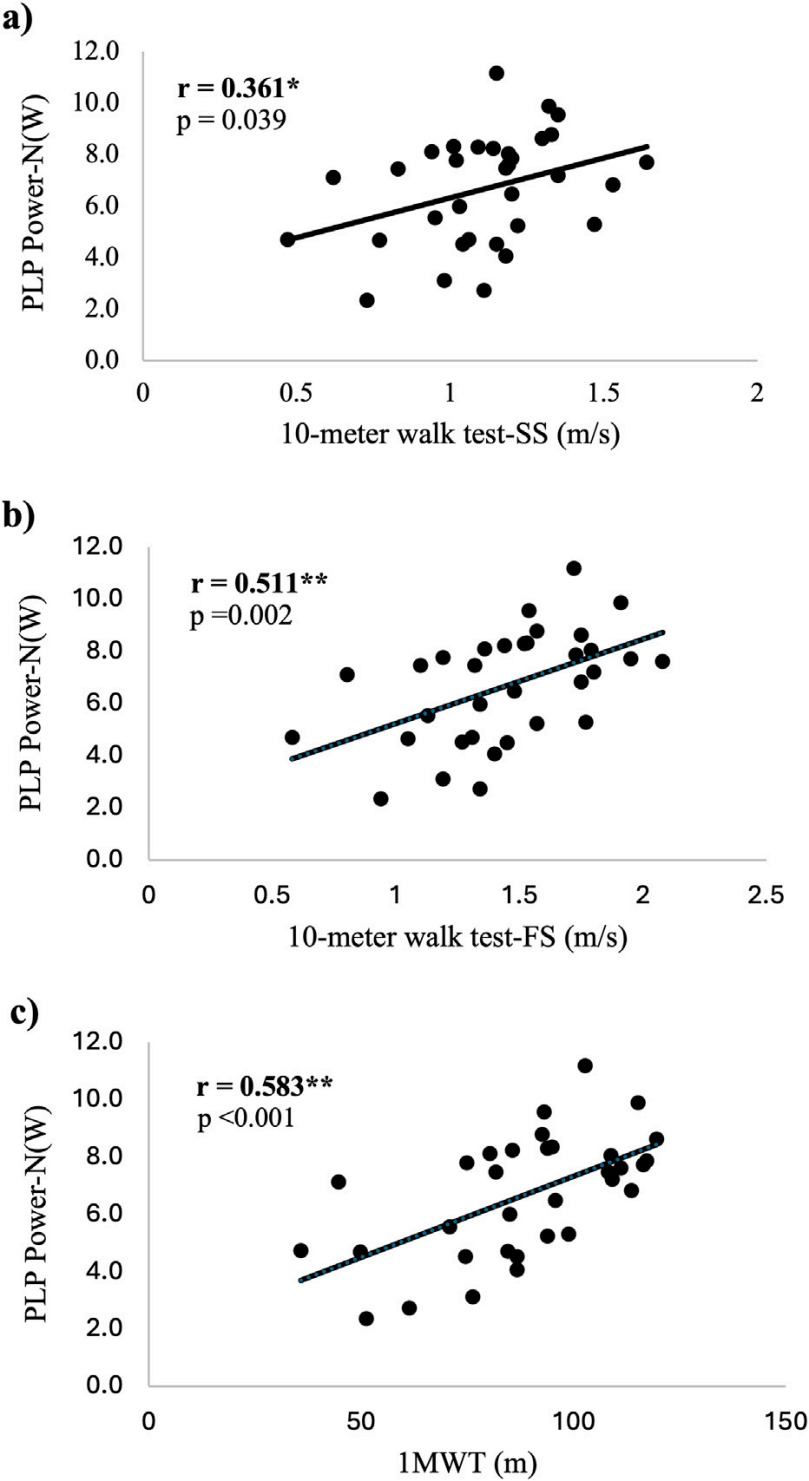
Scatter plots demonstrating the associations between normalized PLP power and tests of walking capacity. **(A)** association between PLP power and SS gait speed **(B)** association between PLP power and FS **(C)** association between PLP power and 1MWT. Abbreviations: PLP Power-N(W) = Power Leg Press Power in watts normalized by body mass; SS = Self-selected; FS = Fast speed; 1MWT = 1 minute walk test; *=statistical significance p<.05; **=statistical significance p<.01; sample size n = 33.

The average load for the PLP test for all participants was 173.6 lbs ±95.5 lbs with a range of 15lbs–410 lbs. This refers to the amount of external weights added to the weight bar, unadjusted for the angle of the sled. The average %1RM across all participants for the PLP test was 47.3% ± 6.8%. PLP peak power was significantly and positively associated with walking speed (SS and FS) and 1MWT (*p* < 0.05) with the strength of the associations ranging from weak to moderate (Figure 1).

### 3.2 Comparison of correlations for associations between PLP and isokinetic tests for measures of walking capacity

**FIGURE 2:**
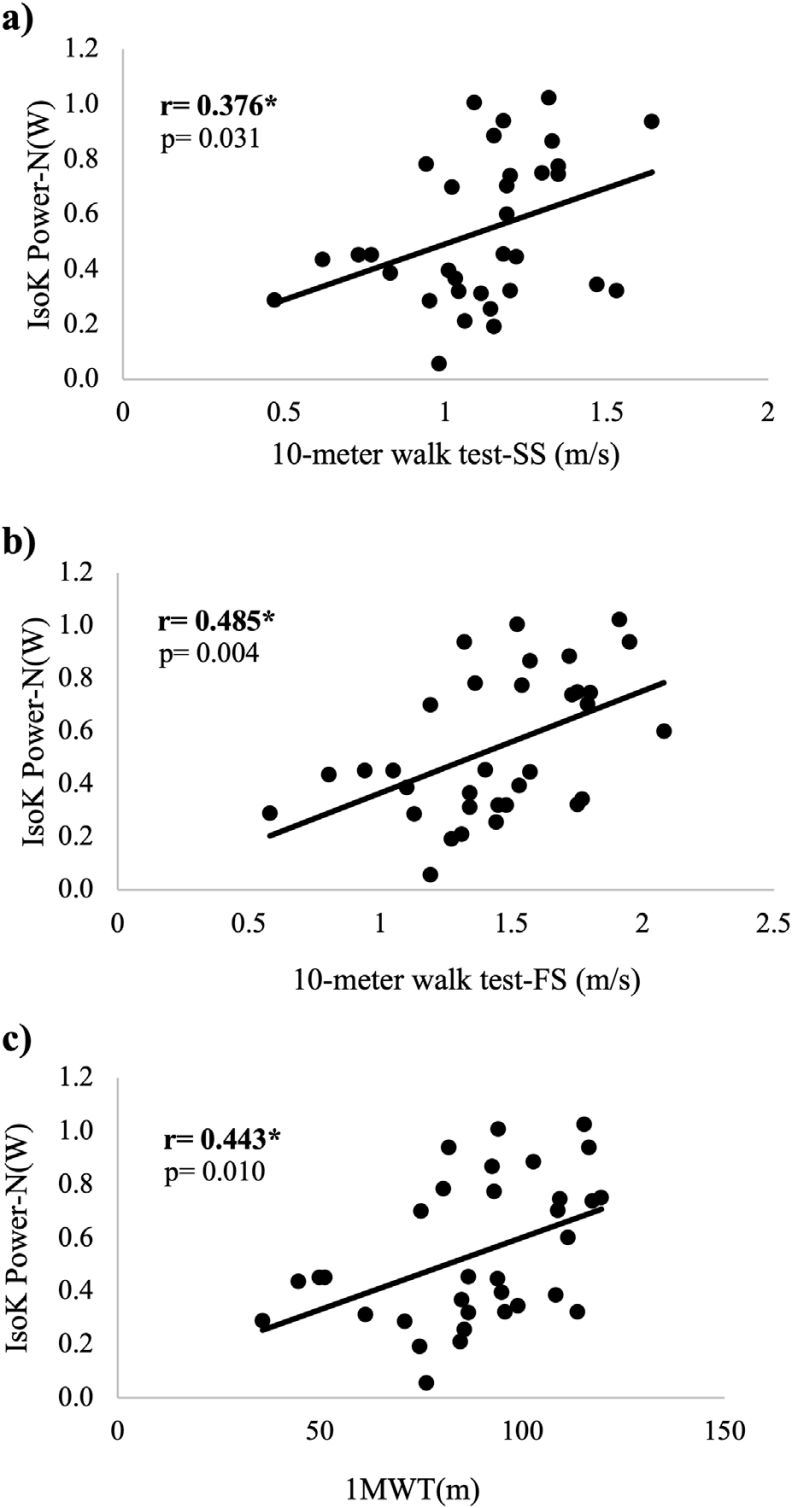
Scatter plots demonstrating the associations between normalized IsoK power and tests of walking capacity. **(A)** association between IsoK power and SS gait speed **(B)** association between IsoK power and FS **(C)** association between IsoK power and 1MWT. Abbreviations: IsoK Power- N(W) = Isokinetic Power in Watts normalized by body mass; SS = Self-selected; FS = Fast speed; 1MWT = 1 minute walk test; *=statistical significance p<.05; **=statistical significance p<.01; sample size n = 33.

There were no significant differences between correlation coefficients for the PLP and IsoK power tests and any of the walking tests (*p* > 0.05 – see Table 2). In addition, scatter plots depicting the associations between PLP power and walking capacity, and IsoK power and walking capacity are displayed in Figures 1, 2, respectively.

**TABLE 2:** Comparison of correlation coefficients for associations between PLP and IsoK Power tests and walking capacity.

Association Tested r _*PLP-walking test*_ */*r _*IsoK-walking test*_	Correlation coefficients
r _PLP-SS speed_/r _IsoK-SS speed_	0.361/.376 *p* = 0.904
r _PLP-FS speed_/r _IsoK-FS speed_	0.511/.485 *p* = 0.822
r _PLP-1MWT_/r _IsoK-1MWT_	0.583/.443 *p* = 0.214

Abbreviations: r *=* Pearson’s r correlation coefficient; PLP- power leg press power; IsoK-isokinetic power; SS- self selected; FS- fast; 1MWT- 1-min Walk Test; sample size n = 33.

### 3.3 Associations between lower extremity power with self-reported activity (performance) and participation

PLP Power was significantly associated with the composite score as well as each subcomponent of the ASKp (*p* < 0.05). All components of the ASKp were moderately correlated with PLP power (r = 0.484–0.690, *p* < 0.05). The PROMIS domains of physical function and mobility, fatigue and peer relationships were moderately correlated PLP power (r = −0.524 to 0.588, *p* < 0.05), while pain intensity demonstrated a weak correlation with PLP power (−0.257, *p* < 0.05). All components of the GOAL were significantly correlated with PLP power (r = 0.361–0.705, *p* < 0.05) except Body Image and Pain (*p* > 0.05). The strength of the correlations for the components of the GOAL ranged from weak to strong. Correlation coefficients between PLP power and all self-reported questionnaires can be found in Table 3.

**TABLE 3:** Correlation coefficients between self-reported activity (performance) and participation and PLP Power in individuals with CP.

Self-report outcome measure	Correlation coefficient with PLP power
ASKp-Composite	0.690**
ASKp-Physical Component	0.484**
ASKp-Locomotion	0.636**
ASKp-Standing Skills	0.521**
ASKp-Transfers	0.547**
ASKp-Play	0.480**
ASKp-Dressing	0.590**
ASKp-Other	0.463**
PROMIS-Physical Function Mobility	0.588**
PROMIS-Fatigue	-.524**
PROMIS-Anxiety	-.268
PROMIS-Depression	-.180
PROMIS-Peer Relationships	0.457**
PROMIS-Pain Interference	-.333
PROMIS-Pain Intensity	-.257*
GOAL-Composite	0.577**
GOAL-ADL	0.478**
GOAL-Gait and Mobility	0.705**
GOAL-Physical Activity and Sport	0.495**
GOAL- Pain	0.243
GOAL- Gait Pattern and Appearance	0.361*
GOAL- Body Image	0.147

Abbreviations: PLP- power leg press; ASKp-Activities Scale for Kids-performance version; PROMIS- Patient-Reported Outcomes Measurement Information System; GOAL- gait outcomes assessment list; ADL-activities of daily living; * = statistical significance *p* < .05; ** = statistical significance *p* < .01; sample size n = 33.

## 4 Discussion

This study aimed to 1) explore the associations between lower extremity muscle power during a PLP test with walking capacity and self-reported walking performance and participation in individuals with CP, and 2) to compare the strength of the associations between two different tests of muscle power and walking capacity. Lower extremity muscle power was significantly related to walking capacity and self-reported walking performance and participation in ambulatory individuals with CP which supports our first hypothesis. Both PLP and IsoK power were significantly related to all three tests of walking capacity. There was no statistically significant difference in the associations of muscle power with walking capacity between the two power tests which refutes our second hypothesis. However, the strength of the associations between power and FS speed and the 1MWT were moderate for PLP power and weak for IsoK power.

### 4.1 Associations between lower extremity power and walking capacity

Walking capacity was significantly correlated with peak power produced during a PLP test in ambulatory individuals with CP, which is consistent with our primary hypothesis. Both the 1MWT and FS speed had stronger associations with peak power (moderate associations: r = 0.583, r = 0.511; *p* < 0.01) compared to SS speed (weak association: r = 0.361, *p* < 0.05) (Figure 1). One consideration for the stronger relationship with 1MWT and FS speed compared to SS speed is the participant’s effort to walk “as fast as possible” for these fast walk tests. This effort is similar to the PLP test which asked individuals to press “as fast as possible” through a squat-like motion in order to promote power generation. Conversely the SS speed asks participants to walk at their usual pace. The similarities in effort of performance and velocity of movement between the PLP and both fast walking tests (FS speed and 1MWT) may explain the stronger associations we reported with these tests as compared to the correlation between SS speed and peak power. The 1MWT evaluates the farthest distance walked in 1 minute at the fastest speed possible. Compared to the other three walking tests in this study, the 1MWT best reflects the components of power (force and velocity) and thus, demonstrated the strongest relationship to power (r = 0.583, *p* < 0.01).

### 4.2 Comparison of correlations for associations between PLP and isokinetic tests for measures of walking capacity

The goal of this secondary analysis was to compare the strength in associations between a newer test of power, the PLP test, and a gold standard measure of power, the IsoK test. Both power tests (PLP and IsoK) were significantly and positively correlated to all measures of walking capacity (SS and FS speed; 1MWT) (Figures 1, 2, respectively). When we compared the strength of the associations between the two tests, there were no significant differences (Table 2). One retrospective study explored the associations between isokinetic average power and walking capacity in individuals with CP (Moreau and Lieber, 2022), and the strength of these relationships was stronger than our data for both tests of power. A moderate to good relationship between isokinetic knee extensor power at 60°/second and both FS gait speed (r = 0.65; *p* < 0.001) and the 1MWT (r = 0.79; *p* < 0.001) was reported in this larger cohort of ambulatory individuals with CP (Moreau and Lieber, 2022). In addition, the authors reported a moderate relationship between SS gait speed and isokinetic knee extensor power (r = 0.59; *p* < 0.01). There are no previously published studies exploring the associations between leg press power and walking capacity in individuals with CP for comparison. The differences in the strength of the correlations between the studies may, in part, be explained by the larger sample (n = 66) and narrower age range (5–25 years) (Moreau and Lieber, 2022) than our study (n = 33; age range = 10–37 years), and different methodologies, particularly for the PLP test. However, we recommend the PLP test for clinical testing of lower extremity power in individuals with CP because it involves multiple muscle groups in a closed chain fashion, is more cost-effective compared to an IsoK testing device, does not constrain the velocity as in isokinetic testing, and is conducted with equipment that is familiar to most practicing clinicians (Pontiff et al., 2023).

### 4.3 Associations between lower extremity power with self-reported activity (performance) and participation

Lower extremity peak power was moderately related to most self-reported measures of mobility-based performance and participation in ambulatory individuals with CP. All components of the ASKp and most components of the GOAL, including the composite scores, demonstrated moderate to strong relationships with lower extremity power. Of clinical significance was the strong relationship between power and the GOAL-Gait and Mobility (r = 0.705, *p* < 0.01, Table 3) and the moderate association with ASKp-Locomotion (r = 0.636; *p* < 0.01, Table 3) and the PROMIS Physical Function Mobility (r = 0.588; *p* < 0.01, Table 3). Moderate associations were also reported between PLP peak power and the ASKp- Transfers, standing skills, and dressing subscales (r = 0.521–0.590; *p* < 0.01, Table 3). The strength of these associations may be explained by the rapid movements required to walk and complete other mobility activities like stair climbing, transfers, and walking. Knee extensor angular velocities range from 186–230°/second for stair climbing and sit to stand tasks (Schenkman et al., 1996; Hortobágyi et al., 2003), and 257–357°/second during terminal swing of walking (Mentiplay et al., 2018), emphasizing the need for velocity with these types of mobility activities. In addition, Van Vulpen et al. demonstrated significant improvements in self-reported mobility on both the Goal Attainment Scaling (GAS) measure as well as the parent-reported mobility questionnaire (MobQues) following functional power training in children with CP (van Vulpen et al., 2018). Therefore, lower extremity power may be a key element in performance of gait and other critical mobility activities in those with CP.

All domains of the PROMIS were significantly correlated with peak power except for scales related to pain interference, anxiety, and depression (Table 3). Anxiety and depression are more prevalent in those with CP than TD peers and both have shown some associations to physical activity (Whitney et al., 2019). However, a variety of physical and non-physical risk factors have been identified as contributors to anxiety and depression in those with CP including: sleep, developmental comorbidities, communication problems, pain, problems with social development, fatigue, general physical activity and mobility restrictions (Whitney et al., 2019). Therefore, the multifactorial nature of these psychological constructs may explain the lack of association with peak power and these domains of the PROMIS. Pain interference is a complex construct that is influenced by physical, structural, cognitive, and psychological elements in those with CP (McKinnon et al., 2019). While pain has been documented to frequently interfere with self-care, sleep, quality of life, and attention (Ramstad et al., 2011; Ostojic et al., 2020), little is known about how pain interference influences specific muscle performance measures like strength and power (McKinnon et al., 2019). Studies reporting frequent pain interference with sleep, self-care, and quality of life in individuals with CP reported moderate pain intensity levels (Ramstad et al., 2011; Ostojic et al., 2020). When we examine our study sample, the average pain intensity for our cohort was 2.7 ± 3.1 with 14 of 33 individuals reporting 0 pain in the past 7 days and the average pain interference score was 48.1 ± 12.2. The low pain intensity and lower than average pain interference scores may partly explain why pain interference was not significantly associated with peak power.

The peer relations, fatigue, and pain intensity domains of the PROMIS were also significantly related to PLP peak power (Table 3). Lower levels of fatigue and pain have been associated with higher levels of walking ability and function in those with CP (Opheim et al., 2009). There was also a moderate association between power and peer relationships. Despite limited published work, impaired walking ability has been linked to reduced social support and peer relationships in children and adolescents with CP (Colver et al., 2015). This evidence suggests there is some relationship between physical performance and relationships with peers in those with CP. Thus, individuals with CP who have greater deficits in muscle performance may perceive lower levels of peer acceptance and quality of friendships.

### 4.4 Limitations

There are several limitations that should be acknowledged in this study. First, the self-reported questionnaires used in this study are designed and validated in children with CP. While children were the majority of the sample in this study, 10 adults with CP participated in the study. Despite this limitation, we still believe that the information collected was valid as the questionnaires focused on mobility tasks that are relevant for both adults and children with CP. For example, when we examine the Gait and Mobility sub-section of the GOAL, which was strongly correlated to power, participants were asked about the difficulty with getting around their home, walking for more than 15 min, going up and down stairs, and walking faster than usual to keep up with others. These are critical daily tasks for adults with CP, and the associations with power may give us important information about how this element of muscle performance impacts activities of daily living in adults and youth with CP. Secondly, our sample included individuals with CP with different sexes and a wide age range. While a heterogenous sample can limit conclusions about more homogenous subgroups of people with CP, we sought to recruit a diverse sample so that the findings can be more generalizable to a larger group of individuals with CP. Third, impairments in posture, dynamic balance, and motor control are known to influence the ability to walk in individuals with CP (Rethwilm et al., 2021). The focus of this study was on the influence of muscle power on walking capacity; however, the impacts of balance and motor control should be evaluated in future investigations. Lastly, this study evaluated correlations between power, walking capacity and self-reported performance and participation. Correlation studies can describe associations or relationships between variables but cannot determine cause and effect. Despite the inherent limitations of correlational studies, these data may help to inform more robust randomized clinical trials aimed at determining the effectiveness of an intervention targeting lower extremity muscle power in improving walking activity and participation outcome measures.

## 5 Conclusion

This study examined the associations between lower extremity power produced during a closed chain leg press task with walking activity and participation in ambulatory individuals with CP. Our findings demonstrate that leg press power is significantly and positively associated with walking capacity and self-reported walking performance and mobility-based participation in ambulatory individuals with CP. For measures of walking capacity, the distance walked in 1 minute (1MWT) and FS walking speed had the strongest associations with PLP power. These fast walking tests may be more strongly linked to peak power generation as they utilize both components of power–force and velocity. Further, muscle power was significantly related to walking capacity in individuals with CP regardless of which method was used to measure muscle power. However, we recommend the PLP test as it incorporates multiple lower extremity muscle groups and is more functional and cost-effective compared to isokinetic dynamometry. Self-reported mobility performance and physical activity outcomes also showed moderate to strong relationships with lower extremity muscle power in subscales involving activities that require more rapid movements, such as gait, transfers, and physical activity. Overall, these findings highlight the critical associations between lower extremity power with walking capacity and mobility-based performance and participation, providing evidence of a strong link between decreased muscle power generation and walking limitations in individuals with CP. Based on the findings of this study, we recommend that clinicians include measures of lower extremity power in their clinical evaluations as power is related to meaningful measures of activity and participation in those with CP. The significant associations between power with walking capacity and self-reported participation highlight activity limitations and participation restrictions that may improve with interventions targeting muscle power generation in ambulatory individuals with CP. The effectiveness of power training interventions on walking capacity, performance, and mobility-based participation should be evaluated in future work.

## Data Availability

The raw data supporting the conclusions of this article will be made available by the authors, without undue reservation.
